# Scalpel Please! A Scoping Review Dissecting the Factors and Influences on Professional Identity Development of Trainees Within Surgical Programs

**DOI:** 10.7759/cureus.20105

**Published:** 2021-12-02

**Authors:** Vasileios Gkiousias

**Affiliations:** 1 Medical Education, East Surrey Hospital, Redhill, GBR

**Keywords:** identity, surgery, surgical training, professional identity formation, medical education

## Abstract

Professional identity development is a multifaceted process that has recently sparked interest in medical education. Literature in professional identity development has focused predominantly on medical students and postgraduate medical training and much less on surgery, despite the unique challenges faced by surgical trainees while trying to emulate the identity of a self-actualized surgeon.

A scoping review was performed to explore the factors and influences on professional identity development of surgeons in training. MEDLINE, PubMed, and OpenGrey databases were initially screened for relevant existing literature of professional identity development in surgical trainees, including quantitative, qualitative, and gray literature, followed by a hand search of references that appeared to be of pertinence. Seven hundred and five records were initially identified. Following the removal of duplicates and application of study selection criteria, 11 studies were included in the review.

Professional identity development in surgical trainees was found to be influenced by a variety of intricately interrelating factors. Gaps in the literature were identified, highlighting possible areas of future research to better elucidate the overall process of identity development in budding surgeons and help guide interventions and programs to facilitate the transition of trainees to qualified and independently practicing surgeons.

## Introduction and background

Professional identity includes the development of professional aspirations, values, and actions [1], as a result of personal reflections and interactions with one’s workplace and the wider society [2]. From an ethical standpoint, assimilating professional ethics as part of one’s identity facilitates a better internal professional regulation [3]. It has been argued that professional identity adds an additional level to Miller’s pyramid from “does” to “is” as individuals come to think, act, and feel as physicians, while also integrating their personal values, attitudes, and beliefs [1]. Nonetheless, this process is far from smooth or straightforward and despite the complexities associated with professional identity development in medicine, little attention had been paid in the literature until recent years, an issue brought to the forefront by the Carnegie Foundation report [1]. This has also been reflected by a shift in medical education literature discourse from professionalism teaching to professional identity nurturing [4].

Literature in surgical education has predominantly analyzed the development of the professional identity of surgeons from a psychological standpoint, often focusing on fixed characteristics and traits that make up the “surgical persona” that is typically characterized by a stoic ethos [5] emphasizing decisiveness, certainty, confidence, and rapid decision-making [6]. As theories on identity development have shifted away from fixed individual traits, so has amalgamating the professional identity of a surgeon, with increasing attention being paid to the social environment and external factors [6].

The recent remodeling of the NHS, alongside mounting political and social pressures and calls for increased accountability, transparency, and safety, has directly impacted medical roles and duties [7]. The European Working Time Directive, implemented in 1998, has created a gap in the provision of surgical services with the proportion of cases performed by surgical trainees having decreased [7]. This discrepancy has been further exacerbated by the increasing complexity of surgical patients and presentations requiring the presence of skilled surgical assistants [8] who are becoming less available. Shorter clinical attachments and higher staff turnover have also been the result of such reforms [9], with loss in continuity of care and breakdown of the traditional dyadic apprenticeship model between trainees and consultants [10]. In a similar manner, duty hour regulations that came into effect in 2003 in the United States, by the Accreditation Council for Graduate Medical Education, have resulted in surgical trainees being required to reach the same standards of cognitive skills and manual dexterity in a much shorter space of time than their predecessors, altering the traditional socialization process in surgery and creating a divide between older generations of surgeons and younger trainees [5].

Lastly, the impact of the COVID-19 global pandemic on surgical training, which has been profound and with unclear consequences for the future progression and careers of current trainees, must be taken into account [11]. Elective surgery had been previously markedly reduced and several trainees had been temporarily redeployed to different and unfamiliar clinical environments, often in Medicine and Intensive Care [11].

Rationale

The literature of professional identity development has approached the process of how one transforms from a layperson to a clinician via different paradigms and theories. However, such perspectives have not been brought together into a unified framework [12] and the focus has been predominantly on undergraduate medical education and seldom on surgical trainees. Given the above and the fact that residency or training is a crucial and formative period of crystalizing the identity of a trainee as a surgeon [5], a scoping review of the literature is deemed necessary, aiming to collate all the aspects of professional identity development in surgical trainees. This review also attempts to elucidate potential gaps or areas that have received less attention within the particular field of study.

Aim

The review question that this scoping review will aim to address is: Which factors or influences affect the process of professional identity development in surgical trainees?

## Review

Methods

Sources of Information

MEDLINE, PubMed, and OpenGrey databases were initially screened for relevant existing literature of professional identity development in surgical trainees, including quantitative, qualitative, and gray literature, followed by a hand search of references that appeared to be of pertinence.

Search Strategy

**Figure 1:**
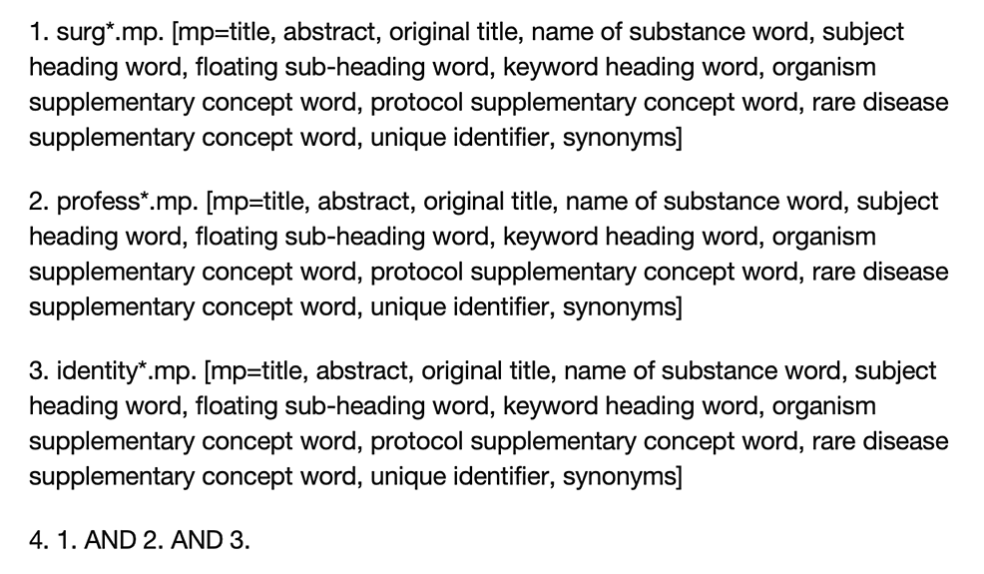
MeSH terms, keywords, and search criteria used for database searches performed MeSH: Medical Subject Headings

A combination of MeSH terms, keywords, and search criteria was imported onto the MEDLINE, PubMed, and OpenGrey databases (Figure 1). 

In an attempt to capture a broader number of studies performed during different time periods, no chronological limits were applied, reflecting wider societal and training program structure changes that could influence the professional identity development of surgical trainees.

Study Eligibility Criteria

Seeking to capture all the relevant literature on the factors affecting or influencing professional identity development in trainees within surgical programs, the inclusion criteria were kept broad: (a) Studies focusing on surgical trainees: any practitioner in a surgical training program, ranging from junior (senior house officers, interns) to senior grades (registrars, residents); (b) Studies focusing on any surgical specialty; (c) Studies with reference to or association with “professional identity development” and potential factors or influences shaping the professional identity of surgical trainees; (d) All study designs and methodologies; (e) Studies from any country; and (g) Studies published in English.

The exclusion criteria were: (a) Studies exploring professional identity development in other healthcare professionals, such as dentists, veterinarians, physiotherapists, occupational therapists, nurses, advanced nursing practitioners, physician associates, social workers, and midwives; (b) Studies focusing on students or undergraduate programs or conversely, fully qualified physicians; (c) Studies focusing on medical specialties or general practice; (d) Studies with only available abstracts; (e) Studies with full text not focusing on factors influencing professional identity development of surgical trainees; and (f) Studies published in languages other than English

Study Selection Process

The available literature was initially screened by title. The next step involved obtaining access to the corresponding articles and screening the abstracts for studies identified as relevant to the aims of this scoping review. The full text was screened in the event that there was a lack of clarity with regards to whether a study met the criteria for inclusion.

Data Extraction

We collected and charted information such as study authors, year, country, participants, methodology, themes or factors influencing professional identity development in surgical trainees, and recommendations on facilitating the process of identity development following identification of studies appropriate for further analysis as part of this review (Table 1). 

**Table 1:** Characteristics of included studies OMF: oral and maxillofacial; SpR: specialist registrar; OR: operating room; F: female; M: male

Author(s)	Year	Country	Participants	Methodology	Themes/factors influencing professional identity development	Recommendations
Aase et al. [13]	2008	Norway	10 trainees and qualified clinicians, including individuals from Cardiothoracic Surgery	Semi-structured interviews	Coping with death, vulnerable responsibility, making a meaningful difference, being part of supporting community, relational fragility.	-Creating an environment of generosity and diversity -Group sessions for discussion/debriefing
Blackburn and Nestel [14]	2014	United Kingdom	Eight Pediatric Surgical trainees	Semi-structured interviews	Themes of troublesomeness: acquiring knowledge of the specialty, developing clinical judgment, acquisition of technical skills, transitions between roles from junior to more senior (with increased perception of responsibility, technical skills, demands of looking after children, managerial duties for more senior roles), validation and self-belief, negative experiences with “emotional scarring” that actually had a positive learning impact through reflection.	-Maximizing educational benefit of negative experiences (e.g., peer group discussions away from M&M environments) -Developing longstanding relationships with mentors/supervisors -Less focus on curricular targets
Cope et al. [15]	2017	United Kingdom	16 General Surgical trainees and qualified surgeons	Semi-structured interviews, field notes, audio and video recordings	Perfectionism, accountability and patient service, self-management and personal resilience, self-critique and seemingly neurotic behaviors (repeated checking on patients, blaming colleagues and external factors for difficulties encountered), teamwork (learning from observing how seniors interact with theater staff), personal initiative and leadership (ensuring resources and efficiency are maximized, filling in gaps where necessary, planning ahead and anticipating).	- Educators need to be able to capture learner attention, increase motivation to learn, and continue to provide external motivators to encourage sustained behavior change - Reinforcement and positive feedback -Apprenticeship model
de Montbrun et al. [16]	2018	Canada	13 trainees from Cardiothoracic surgery, Neurosurgery, Pediatric surgery, Orthopedics, General Surgery and Gynecology	Semi-structured interviews	“Getting undressed,” feeling “exposed and vulnerable,” “suiting up,” and “tailoring the fit.”	-Mentorship -Early training programs and interventions to facilitate transitioning to new roles
Lingard et al. [17]	2002	Canada	52 operating theater team members, including nine surgical trainees	Focus group interviews	﻿﻿Subjects’ discursive constructions of other team members and their motives were generally inconsistent with those members’ self-perception. ﻿The phenomenon of dissonant discursive construction may prove particularly problematic for novices, who possess fewer experiential insights into the team dynamic and are thus at greater risk of misreading the story unfolding before them in the OR. ﻿Their ‘legitimate peripheral participation’ extends beyond the explicit domains of surgical, anesthetic, or nursing techniques and into the often implicit realm of interprofessional relations.	-Emphasis on communicative exchanges between different team members particularly in moments of tension
Myers et al. [18]	2018	United States	42 General Surgical trainees (24 M:18 F)	Semi-structured interviews	Regard for professional titles (with female trainees being more frequently disregarded both by patients and physicians), ﻿ perceptions, attitudes and gender-specific disadvantages (with female trainees being perceived more as lacking authority or being aggressive conversely if exhibiting more dynamic behaviors, less confident, more at risk of receiving unprofessional remarks, pressured to participate in unprofessional behaviors, lack of mentorship, difficulty completing tasks due to pressure), reduced self-worth.	-
Tahim [19]	2015	United Kingdom	Seven Maxillofacial Surgical trainees	Semi-structured interviews	1) Elements common to all OMF surgeons: sustained enjoyment, facial aesthetics, regional expertise, rejection of other specialties; 2) Differentiating elements: dual qualification, expert knowledge, technical skills, junior workforce supervision (being more hands-on as a SpR), low specialty profile; 3) Ideal qualities and attributes: technical skills, leadership, role-modeling, attitude toward others.	-
Veazey Brooks and Bosk [5]	2012	United States	﻿One group of 13 surgical residents, one group of 10 surgical interns, and one group of 15 senior surgical residents	Field observations, semi-structured interviews	Neutralizing: dealing with higher levels of complexities and comorbidities, higher work intensity, higher efficiency embracing: improved quality of life, improved recruitment and retention in surgery, focus on work-life balance anxious: less opportunity to achieve same standards of practice as older cohorts, doubts about own skills and practice and concerns about perceptions of seniors.	-
Quinn et al. [20]	2014	Ireland	﻿Members of a General Surgery department, including 12 senior trainees (registrars) and 14 junior trainees (senior house officers and interns)	Video recordings and semi-structured interviews	Hierarchy legitimacy of participation in a community of practice.	﻿-Creating a less threatening, more egalitarian environment with defined opportunities for juniors to contribute -Senior involvement, guidance, and feedback
Lipsman, Khan and Kulkarni [21]	2017	United States	Surgical trainees of different grades	Opinion paper	Junior trainees: trust vs. mistrust, autonomy vs. doubt, initiative vs. complacency Intermediate trainees: identity vs. role confusion, generativity vs stagnation; Senior trainees: authoritarian vs. authoritative, industry vs. inferiority, ego integrity vs. despair.	-Supervision and meetings with discussion and objectives tailored to different developmental stages and needs
Allen et al. [22]	2019	Canada	Surgical trainees of various different grades	Scoping literature review	Autonomy.	-Meaningful feedback to optimize educational impact of autonomous practice

Results and discussion

Database Search Results

**Figure 2:**
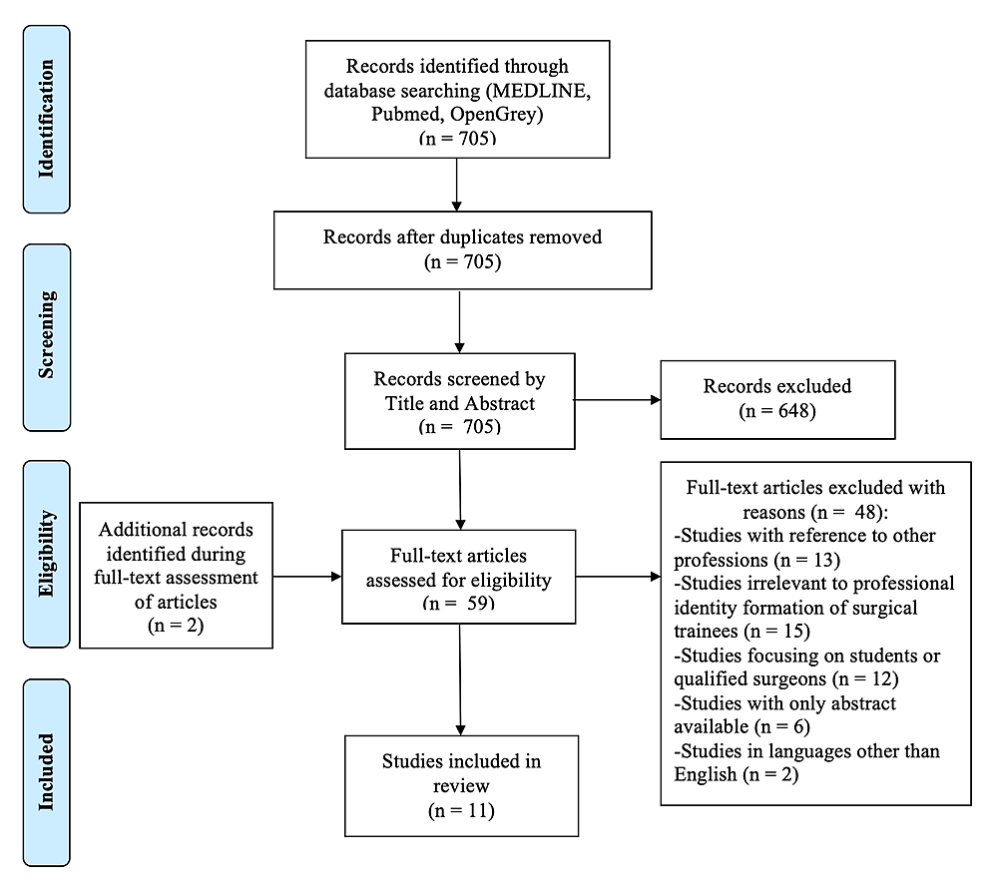
Flow diagram detailing process of final study selection for inclusion in the scoping review

The MEDLINE, PubMed, and OpenGrey databases, along with the hand-search generated 705 articles. Of these, 648 articles following title and abstract screening were excluded, while the remaining 57 articles were reviewed by their full text. Along the process, two additional articles were identified as being of potential interest from the bibliography of the full-text screened articles, increasing the total number to 59. After a full-text review of the 59 articles and in conjunction with the inclusion and exclusion criteria set for this scoping review, professional identity development and relevant influences in surgical trainees were only explored by 11 articles, which were included in the final analysis (Figure 2). 

Characteristics of Included Studies

The included studies (Table 1) were published between 2002 and 2019. The study designs comprised predominantly original qualitative research, one theoretical paper, and one literature review. Articles presented data from clinical environments in Norway, the United States, Canada, the United Kingdom, and Ireland.

**Figure 3:**
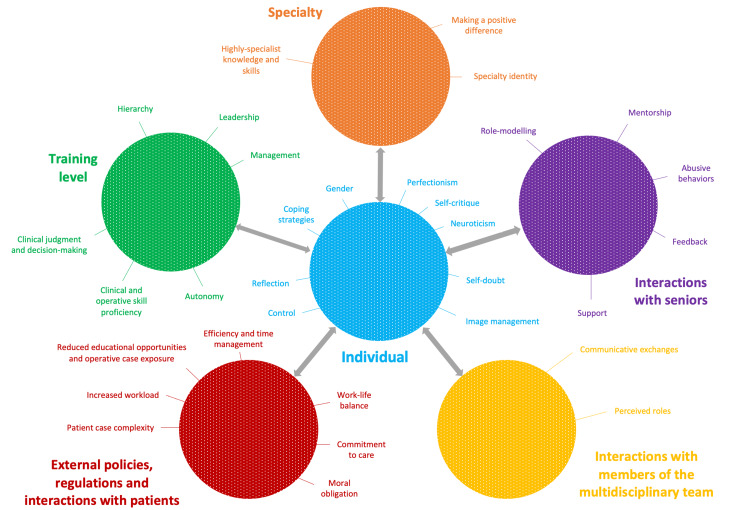
Factors and influences shaping the professional identity of surgical trainees

Figure 3 depicts the identified themes from the literature under examination for this thesis. It should be noted that all of the aspects are linked and interact with one another to form an intricate web with the surgical trainee placed in the epicenter.

Person-Specific Aspects

Among surgical trainees, traits such as perfectionism, self-critique and neuroticism, self-doubt, the need for self-management and control, including not only of physical responses but also of a projected image, as well as developing resilience and coping strategies were found to be common. These attributes might not necessarily be inherent in an individual, but might be learned through the course of their surgical training, as a result of being embedded in a community of practice and assimilating behaviors and beliefs of more senior, core members of that community [15].

Image management is of particular interest in surgery as it can create identity dissonance, particularly during challenging moments such as when operating or dealing with a difficult case. Conflict arises between internalized views of one’s self faced with uncertainty and doubt versus wanting to project an image of certitude in response to external pressures from the immediate environment [6]. Although this appears problematic for one’s professional identity development, the pressures to “measure up” can also be positive, by promoting surgeons’ self-efficacy, believing in themselves that they can be successful despite difficulties. This, therefore, creates a positive feedback loop that in turn leads to better performance and resilience in the face of adversity [6]. Trainees ultimately emulate the very identity of surgeons that they project [6].

Coping mechanisms in response to challenges, pressures, as well as facets such as dealing with mortality and one’s own vulnerability were recognized as important aspects as they have the potential to act as catalysts in the process of professional identity development [1]. Resilience and the ability to recover from stress and challenges have been intrinsically linked to well-being that is in turn strongly linked with having a strong sense of identity [23].

Reflection was also found to hold a key role in professional identity development within the literature examined. Consisting of both metacognitive (“thinking about thinking”) and meta-affective (“feeling about feeling”) components, reflection can help trainees develop their empathy, navigate the complex paths of surgical practice, and offers a moral compass when faced with challenging dilemmas [23].

Gender was identified as another important aspect of professional identity development in trainees, with female individuals found to be at a disadvantage when compared to their male counterparts [18]. Despite the increase in numbers of female trainees in surgical programs, there still exists a glass ceiling effect in surgery, due to traditional gender roles and a more masculine surgical culture, sexism within the clinical environment, and lack of mentorship [24]. The findings included in this scoping review parallel those by a previous study that found that female medical students on surgical rotations had less "workplace affordances", with fewer opportunities and hands-on exposure to surgical procedures [25]. This creates feelings of inadequacy and low self-esteem and worth, adversely affecting the process of identity development as a surgeon [18].

Specialty-Specific Aspects

Being in a position to make a positive difference to patients’ lives and possessing highly-specialized knowledge and skillsets as a result of one’s surgical training, were quoted as positive influences for one’s professional identity as a surgeon [13,19]. Identifying one’s self with the features of a surgical specialty is fundamental for assimilating professional identity; however, as has been posited, this might be less straightforward for some specialties [19]. Oral and Maxillofacial Surgery (OMFS) was found to struggle with defining its identity as a specialty [26], as it integrates elements from surgery and dentistry, with alternative career pathways that can be followed to qualification, often not clearly known or understood by other specialties and healthcare providers. Consequently, this identity confusion within the specialty itself can have implications for the coherent integration of identity of trainees as surgeons, especially given the duality of their previous training and backgrounds, which in turn have already shaped elements of their identity.

Training Level-Specific Aspects

From a professional identity perspective, progressing through the different levels and stages of one’s surgical training was found to be transformational and often associated with developing clinical and operative skill proficiency, clinical judgment, and decision-making, as well as assuming more managerial and directive positions with greater responsibility [14,16,21]. All three papers that examined the transition of trainees to a higher grade did so from a threshold standpoint [14,16,21]. Trainees were found to be ﻿in a state of liminality [27], moving from one stage to the next, shedding elements of their former professional identities, or “getting undressed” [16], as such elements are no longer compatible with their professional development. This process was also found to be laden with self-doubt, ambiguity, concerns about one’s preparedness for independent practice, and the need for validation. It has been also found previously that such transitions within surgery can be a significant source of anxiety, depression, and burnout [16]. Interestingly, the above aspects were found to be common both within junior and senior surgical trainee cohorts, with senior trainees projecting an image of self-assurance while “suiting up”, despite internal concerns about their preparedness [16]. This highlights the need for a supportive network within a surgical community of practice, throughout the span of one’s career.

Transitioning to a higher level of training is correlated with higher levels of autonomy, namely in terms of operative skills, which are of paramount importance in surgery. Increased autonomy was found to be associated with increased confidence, preparedness, and better decision-making, as well as acting as a facilitator for professional identity development [22]. However, it has been highlighted that there needs to be a balance between the need for autonomy and demands for supervision and maintaining a high level of patient safety [28].

Hierarchy was an aspect identified predominantly in one study [20], both through the seating arrangements of the members of a General Surgical department and also through the degree of participation in discussion, during a journal club session. More senior trainees were found to be more engaged and actively contributing, whereas more junior trainees were perceived to have less legitimacy, conferring them an identity of marginality [20]. This demonstrated that even though members of the same community might be participating in the same activities and accessing the same resources and pool of knowledge, their professional identity process is not equally benefited.

Interactions with Members of the Multidisciplinary Team

The effect of inter-group communicative exchanges among different operating theater team members, as well as their interpretation by the different individuals, also focusing on their impact on professional identity development for novices was addressed by one study [17]. Language was highlighted as playing a catalytic role in the process, being a social act with both descriptive and constructive potential for one’s reality and identity [17]. More junior trainees were found to have created oversimplified and dissonant constructions of other team members’ motivations, values, and roles, often as a result of echoing the perceptions of their seniors via observed discourses [17]. This was perceived as an adaptive mechanism of aligning one’s professional identity with that of their role models, as an attempt of legitimizing their peripheral participation in the particular community of practice and obtaining the desired intragroup membership, at the expense of interprofessional identification, effective collaboration and equally crucially, the development of an intricate professional identity [17]. The authors also emphasized that this might also be the result of education and training of different individuals in uni-professional silos with limited understanding of others’ roles, functions, skillset,s and backgrounds, who are however expected to work coherently as a team [17], such as is the case within surgery. This reflects a wider discrepancy between actual practice and educational aspiration for interprofessional practice and collaboration relying on a distributed agency, whereby there is mutual understanding and respect for different professional roles [29]. Linguistic exchanges and professional identity also interact in a bidirectional manner, as possessing a complex identity can permit individuals to communicate in a less distant and more inclusive manner, inspiring trust and acceptance not only among colleagues but also with patients, resulting in better healthcare provision and outcomes [3].

External Policies, Regulations, and Interactions with Patients

Externally imposed policies and regulations were brought into effect as a result of concerns about the performance of trainees and patient safety due to working long hours and associated fatigue and sleep deprivation, poor ability to sustain concentration and engage in complex decision-making processes, and impaired fine motor control and risk of injury to self and others while driving after long shifts [5]. The blanket application of such policies affected all specialties, but its impact potentially affected surgery the most, given the overall process of socialization of training surgeons changed and became markedly different from the traditional rites of passage encountered by older generations of surgeons, encompassing a stoic ethos, which has been closely related to long working hours and persevering under adverse conditions [5]. Traditionally, succeeding in spite of such circumstances provided trainees with the assurance that they could overcome any challenge, which has been thought of as an essential part of an actualized surgical professional identity [5]. While these changes in working hour patterns were viewed as negative by trainees and more senior consultants, due to concerns about exposure to cases, obtaining the necessary operative and clinical skills, and doubt regarding whether one possesses the necessary resilience to progress with their surgical training, it was also discovered that the particular changes were viewed as neutral or even positive in some cases [5]. With regards to the former, trainees mentioned that the process of their surgical identity development was unaffected, as working less hours has been counterbalanced by increasing workload, patient case complexity, and need for efficiency and time management in comparison to older cohorts of surgeons [5]. Of particular interest is that the implementation of external regulations was viewed as positive by a group of trainees in terms of promoting a better work-life balance, switching the focus to wellbeing and life outside of surgery with added benefits for the profession itself by improving retention and inviting more well-rounded individuals to the field of surgery [5]. This echoes the fact that professional identities are dynamic and constantly evolving in response to external stimuli and personal circumstances and beliefs and can result in changes in how the overall surgical identity is collectively shaped within the context of a surgical community, perhaps reflecting a gradual cultural shift in surgery.

Interactions with patients and commitment to providing effective and safe care were also identified as important factors in the process of professional identity development of surgical trainees. This was approached from a moral standpoint demonstrating that there exists a social contract between society and medicine, creating expectations on both sides [4]. In turn, this is closely related to the significance of moral development and moral maturation as part of one’s professional identity [12,30], which as has been found previously, can be a complicated process in its own right, creating conflicts and angst for surgical trainees [31].

Interactions with Seniors

Role-modeling and mentorship were seen as focal points in the interactions between trainees and senior surgeons in the process of professional identity development of the former. The importance of mentoring has been previously highlighted, particularly during transitional points in an individual’s career, with positive effects on confidence, career satisfaction, research prospects, productivity, professional socialization, and the building of collaborative partnerships [32]. Mentors can also promote a sense of belonging and value for their mentees within a professional setting [33]. Moreover, effective mentorship has been found to ameliorate racial and gender inequalities [34]. Conversely, observed negative and abusive behaviors such as workplace bullying can create helplessness and stall the overall development of a trainee’s identity [35], as was observed in the case of female surgical trainees [18]. Cultivating a culture of feedback and support are instrumental in the process and vital for generating confidence and self-esteem in trainees [15]. Moreover, a tailored approach with objectives and goals set for each trainee according to their different developmental stages and needs is required [21].

Gaps in the Literature

The range of studies and their combined findings included in this scoping review undoubtedly contribute to a more complete picture on the factors affecting the process of professional identity development in surgical trainees (Figure 3). Nevertheless, there was a noticeable lack of detail on participant demographics, characteristics, and backgrounds, with the exception of one study focusing on gender differences in the experiences of surgical trainees [18]. This has been previously identified by literature on professional identity development in Medicine and commenting on the exclusion of individuals from more diverse and less mainstream populations, who are likely to experience the process and transitions of professional identity development significantly differently [36]. The ensuing sociocultural bias creates a problematic antithesis when studying the phenomenon of professional identity development, as in its core, the process entails individuals being able to integrate aspects of themselves and backgrounds into a coherent self in tandem with their emerging professional identities [4]. Furthermore, in view of the globalization of modern healthcare, with healthcare professionals being educated and socialized in different countries and striving for membership in new communities of practice within different healthcare systems, more attention should be paid to such personal attributes and beliefs as tensions and conflicts resulting in identity dissonance can become prominent [4]. Finally, the impact of postgraduate examinations, with the associated time, financial and psychological pressures, and the implications for one’s professional identity as a surgeon especially when struggling to progress with their career, also merits further exploration in future research.

Limitations

Given the complexity of the subject under study, fully differentiating which aspects are relevant for trainees at different stages of their career was challenging, as the majority of the reviewed literature often included a mix of seniorities and grades. As such, the map constructed as part of this scoping review (Figure 3) and the respective attention paid to each of the identified aspects provides an overview that might not be universally true or accepted by fellow researchers, readers, and policymakers potentially interested in the subject. What can be also considered as a limitation to this scoping review is the fact that there is no formal appraisal of the quality of research studies included nor a synthesis of the comparative weight of evidence provided from those studies as to which factors appear to be more important when it comes to professional identity development in surgeons, which would perhaps have been better addressed through a systematic review.

## Conclusions

The professional identity development of surgical trainees is a dynamic, highly complex, and iterative process involving an attempt to integrate personal values, beliefs, aspirations, and skills with those of the surgical profession into a coherent self. The influence of one’s social environment and interactions with colleagues, seniors, patients, and policymakers are transformative and can exert both positive and negative effects on budding surgeons. This scoping review aimed to collate the minutiae of professional identity development of surgeons in training into a comprehensive picture and could serve as a roadmap for prospective research, focusing on less thoroughly studied aspects or identified gaps in the literature. Consequently, this could lead to better understanding and facilitating the process of professional identity development for future generations of surgical trainees, ultimately leading to more effective training programs and improved healthcare provision.
